# Diarrhoea in the ICU: respective contribution of feeding and antibiotics

**DOI:** 10.1186/cc12832

**Published:** 2013-07-24

**Authors:** Ronan Thibault, Séverine Graf, Aurélie Clerc, Nathalie Delieuvin, Claudia Paula Heidegger, Claude Pichard

**Affiliations:** 1 Nutrition Unit, Geneva University Hospital, Rue Gabrielle-Perret-Gentil 4, Geneva 14 1211, Switzerland; 2 Division of Intensive Care, Rue Gabrielle-Perret-Gentil 4, 1211 Geneva University Hospital, Geneva, Switzerland

**Keywords:** Tube feeding, Liquid stools, Antifungal drugs, *Clostridium difficile*, Nursing care

## Abstract

**Introduction:**

Diarrhoea is frequently reported in the ICU. Little is known about diarrhoea incidence and the role of the different risk factors alone or in combination. This prospective observational study aims at determining diarrhoea incidence and risk factors in the first 2 weeks of ICU stay, focusing on the respective contribution of feeding, antibiotics, and antifungal drugs.

**Methods:**

Out of 422 patients consecutively admitted into a mixed medical–surgical ICU during a 2-month period, 278 patients were included according to the following criteria: ICU stay >24 hours, no admission diagnosis of gastrointestinal bleeding, and absence of enterostomy or colostomy. Diarrhoea was defined as at least three liquid stools per day. Diarrhoea episodes occurring during the first day in the ICU, related to the use of laxative drugs or *Clostridium difficile* infection, were not analysed. Multivariate and stratified analyses were performed to determine diarrhoea risk factors, and the impact of the combination of enteral nutrition (EN) with antibiotics or antifungal drugs.

**Results:**

A total of 1,595 patient-days were analysed. Diarrhoea was observed in 38 patients (14%) and on 83 patient-days (incidence rate: 5.2 per 100 patient-days). The median day of diarrhoea onset was the sixth day, and 89% of patients had ≤4 diarrhoea days. The incidence of *C. difficile* infection was 0.7%. Diarrhoea risk factors were EN covering >60% of energy target (relative risk = 1.75 (1.02 to 3.01)), antibiotics (relative risk = 3.64 (1.26 to 10.51)) and antifungal drugs (relative risk = 2.79 (1.16 to 6.70)). EN delivery *per se* was not a diarrhoea risk factor. In patients receiving >60% of energy target by EN, diarrhoea risk was increased by the presence of antibiotics (relative risk = 4.8 (2.1 to 13.7)) or antifungal drugs (relative risk = 5.0 (2.8 to 8.7)).

**Conclusion:**

Diarrhoea incidence during the first 2 weeks in a mixed population of patients in a tertiary ICU is 14%. Diarrhoea risk factors are EN covering >60% of energy target, use of antibiotics, and use of antifungal drugs. The combination of EN covering >60% of energy target with antibiotics or antifungal drugs increases the incidence of diarrhoea.

## Introduction

Diarrhoea is often defined as the passage of at least three liquid stools per day [1]. Diarrhoea is frequently observed in ICU patients, but the reported prevalence differs according to the definition and the setting, between 2 and 95% [2-6]. The causal factor of diarrhoea is sometimes obvious; that is, *Clostridium difficile* infection, recent intestinal resection, malabsorptive digestive disease. However, in most cases, diarrhoea is supposed to be multifactorial, without any identified causal factor [7]. Antibiotics, including antifungal drugs, and enteral nutrition (EN) are among the most suspected causal factors of diarrhoea [3,8-10]. However, the role of their combination in the onset of diarrhoea in the ICU is unclear. EN is the first choice for nutritional support in the ICU when the gastrointestinal tract is functional [11]. Some authors argue that EN reduces the incidence of diarrhoea through a better preservation of intestinal trophicity and epithelial intestinal barrier function, while others find a positive relation between EN and diarrhoea [3,8-10]. EN solutions containing fibres are proposed with the aim to control transit and to prevent or treat diarrhoea [12-14]. Diarrhoea is associated with dehydration, impaired electrolyte balance, bedsores, and catheter-related infections, and increases the burden of nursing care and related investigations. In daily clinical practice, therefore, the onset of diarrhoea frequently leads to discontinuation of EN [5], which increases the risk of energy and protein deficit, in turn related to undernutrition and poor clinical outcome [15].

This prospective observational study in a tertiary ICU population therefore aims to determine, during the 14 first days of ICU stay, the incidence of diarrhoea, the timing of diarrhoea onset, and the risk factors for diarrhoea focusing on the respective contribution of feeding, antibiotics, and antifungal drugs.

## Materials and methods

### Patients

**Figure 1:**
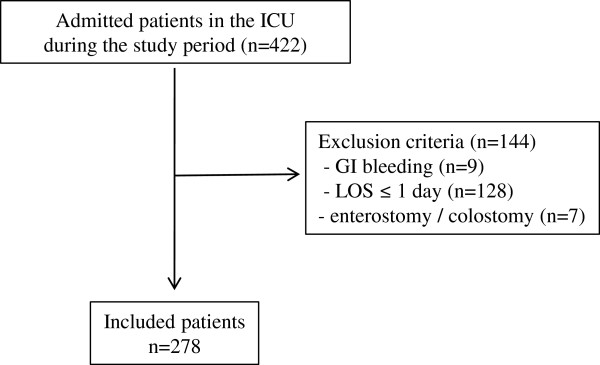
**Flowchart of the study cohort.** GI, gastrointestinal; LOS, length of stay.

This prospective observational study was performed in consecutive mixed medical–surgical patients admitted during a 2-month period to the ICU of a tertiary referral hospital (Geneva University Hospital, Geneva, Switzerland). All adult patients admitted to the ICU staying more than 24 hours, with no admission diagnosis of gastrointestinal bleeding, and without enterostomy or colostomy, were included in the study (Figure 1). Diarrhoea was defined as the elimination of at least three liquid stools per day, and was reported by ICU nurses on the computerised information data management system (CliniSoft 6.2; General Electric, Milwaukee, WI, USA). Because the aim of our study was to evaluate the risk of diarrhoea under the exposure of risk factors in the ICU, the diarrhoea episodes occurring during the 24 first hours following ICU admission were not analysed (*n* = 6 diarrhoea days). Also, diarrhoea occurring during the 48-hour intake of laxative drugs (*n* = 31 diarrhoea days) or the diarrhoea associated with a positive laboratory documentation of *C. difficile* was not analysed (*n* = 4 diarrhoea days), since these are well-known causative factors of diarrhoea. The following parameters were documented at ICU admission: age, gender, weight, height, body mass index (BMI), Acute Physiology and Chronic Health Evaluation II score [16], Simplified Acute Physiology Score (SAPS) II, diagnosis category, and energy target. During the 14 first days in the ICU, we collected the number of liquid stools per day, the use of EN, the amount of energy delivered by EN, and treatments: antibiotics, antiviral, antifungal, laxatives, prokinetics, probiotics [17], and immunosuppressants. The length of stay, mortality, the presence of bedsores, use of invasive and non-invasive ventilations, and renal replacement therapy were collected at the end of the ICU stay.

This study was approved by the ethical committee of Geneva University Hospital as a quality of care control programme (#07-250 (NAC 07–098)), and as such no patient’s written consent was required.

### Feeding strategy

The energy target was defined as recommended by European Society for Parenteral and Enteral Nutrition guidelines [11]; that is, 20 to 25 kcal/kg ideal body weight/day during the 96 first hours of ICU stay, and thereafter 25 to 30 kcal/kg ideal body weight/day for females and males, respectively. Anamnestic body weight was used for patients with BMI ≤20 kg/m^2^. EN was started on day 1 after ICU admission in all patients unable to eat orally at a rate of 20 to 30 ml/hour up to a maximum of 150 ml/hour, and administered continuously according to our routine protocols. EN products consisted of polymeric, fibre-enriched formulas containing: energy, 1.0 to 1.5 kcal/ml; proteins, 16%; lipids, 30 to 35% (medium-chain triglycerides 0 to 6%); and carbohydrates, 49 to 56% of the total provided energy. Fibres consisted of 22 g/l guar gum or 17.6 g/l soluble and nonsoluble fibres mix including 7 g/l fructo-oligosaccharides. EN formulas came from two different manufacturers (Nestle Medical Nutrition, Vevey, VD, Switzerland; Abbott AG, Baar, Zg, Switzerland).

### Statistical analysis

Baseline characteristics are presented as mean ± standard deviation or median (minimum; maximum) for quantitative variables, and as number (percentage) for qualitative variables. The quantitative variables were compared using unpaired Student’s *t* test or the Mann–Whitney test as appropriate. Proportions were compared with a chi-square or Fisher’s test as appropriate. The cohort characteristics were presented as median (minimum; maximum), and per 100 patient-days.

Only the first 14 days after the ICU admission were considered for analysis because our study aims to assess the diarrhoea risk factors during the early phase of the ICU stay. Diarrhoea incidence was analysed as the proportion of patients with at least 1 day of diarrhoea over the total number of patients included, expressed as a percentage, and the number of days with diarrhoea over the total number of analysed days during the study period, expressed per 100 patient-days. Patient-days indicate a unit of time during which the ICU facilities are used by a patient; for example, 50 patients in the ICU for 1 day would represent 50 patient-days. The day of diarrhoea onset was defined as the day of the first diarrhoea. Diarrhoea days occurring during the first 24 hours, in the 48 hours following a laxative treatment, and those in the context of *C. difficile* infection were not analysed. The latter two were excluded since they represent well-known aetiologies of diarrhoea. The risk factors were considered present if observed at least 1 day during the 48 hours before the day with diarrhoea. The variable ‘EN’ was analysed according to the presence/absence of EN and the percentage of energy target coverage ≤60% versus >60%. A cutoff value of 60% was chosen because this is the level of energy coverage (or energy target) below which ICU patients were considered to have an energy deficit [18]. The BMI and SAPS II score were separated according to their median values. The variables ‘antibiotics’, ‘antifungal drugs’, ‘immunosuppressants’, and ‘prokinetics’ were analysed according to their presence or absence. The variable ‘probiotics’ was not analysed because only two patients did receive probiotics.

To account for the correlation among the repeated observations for a given subject, the association between risk factor exposure and diarrhoea was analysed using the Generalized Estimated Equations model with a binomial family, a logit link, and an exchangeable intracorrelation structure [19]. This correlation structure was selected by the quasi-likelihood criterion [20]. The standard error of the parameters was adjusted for clustering on patients. From the fitted model, the estimates of the adjusted risks ratios were obtained with exp{β}, where β is the estimated parameter by the multivariable Generalized Estimated Equations model. The exposure to a risk factor is described as the time at risk expressed in patient-days, and as the incidence rate and estimated relative risk with 95% confidence interval.

A stratified analysis was performed to measure the specific effect of antibiotics and antifungal drugs under EN. Homogeneity assumption was tested using the chi-square test and overall incidence rate ratios were adjusted with Mantel–Haenszel weights [21]. Data analysis was performed using Stata 12.0 software (College Station, TX, USA). The two-sided *P* value was reported and the significant level was <0.05.

## Results

### Characteristics of the included patients

Among the 422 patients admitted to the ICU during the study period (2,038 patient-days), 278 were consecutively included (Figure 1). A total of 1,595 patient-days were analysed after exclusion of diarrhoea days occurring during the first 24 hours in the ICU, of diarrhoea days occurring during the 48 hours following laxative treatment, or of *C. difficile* infection. EN, antibiotics, antifungal drugs, immunosuppressants, and prokinetics were observed during the 48 hours preceding the analysed patient-day in 69%, 62%, 11%, 5%, and 5% of patient-days, respectively. Patients’ characteristics according to the presence or absence of at least 1 day of diarrhoea are shown in Table 1. Those with diarrhoea were characterised by a higher proportion of females, respiratory and gastrointestinal diagnoses, and low BMI. Patients with diarrhoea had a more severe disease: high Acute Physiology and Chronic Health Evaluation II score and SAPS II at admission, high proportion of bedsores, and longer ICU length of stay. Age and medical or surgical types of diagnosis were not significantly different between the two groups.

**Table 1:** Patients’ characteristics according to the presence or absence of diarrhoea

	**Patients with diarrhoea**	**Patients without diarrhoea**	***P *** **value**
	**(n = 38, 14%)**	**(n = 240, 86%)**	
**At admission**			
Age (years)	59 ± 17	59 ± 16	0.839
Gender (male/female)	17(45)/21 (55)	155 (65)/85 (35)	0.019
BMI (kg/m^2^)	23.9 ± 4.3	25.8 ± 4.7	0.029
APACHE II score	25 ± 7	20 ± 8	0.003
SAPS II score	50 ± 17	41 ± 17	0.004
Medical/surgical	25 (66)/13 (34)	143 (60)/97 (40)	0.467
Diagnosis			
Gastrointestinal surgery	4 (11)	7 (3)	0.005
Gastrointestinal disease	2 (5)	4 (2)	0.005
Respiratory	14 (37)	43 (18)	0.005
Neurologic	3 (8)	43 (18)	0.005
Cardiac surgery	3 (8)	30 (12)	0.005
Trauma	2 (5)	18 (8)	0.005
Infections (other than respiratory)	3 (8)	15 (6)	0.005
Cardiac arrest	1 (3)	14 (6)	0.005
Myocardial ischemia	1 (3)	45 (19)	0.005
Other	5 (13)	21 (9)	0.005
**During ICU stay**			
Ventilation			
Invasive	27 (71)	133 (55)	0.070
Non-invasive	8 (21)	34 (14)	0.271
Renal replacement therapy	3 (8)	11 (5)	0.417
Bedsore	10 (26)	12 (5)	<0.001
Length of stay (days)	15 ± 14	6 ± 7	<0.001
Death in the ICU	6 (16)	27 (11)	0.421

Diarrhoea was defined as at least three liquid or soft stools per day. Values are number (%) or mean ± standard deviation. APACHE, Acute Physiology and Chronic Health Evaluation; BMI, body mass index; SAPS, Simplified Acute Physiology Score.

### Epidemiology of diarrhoea

**Figure 2:**
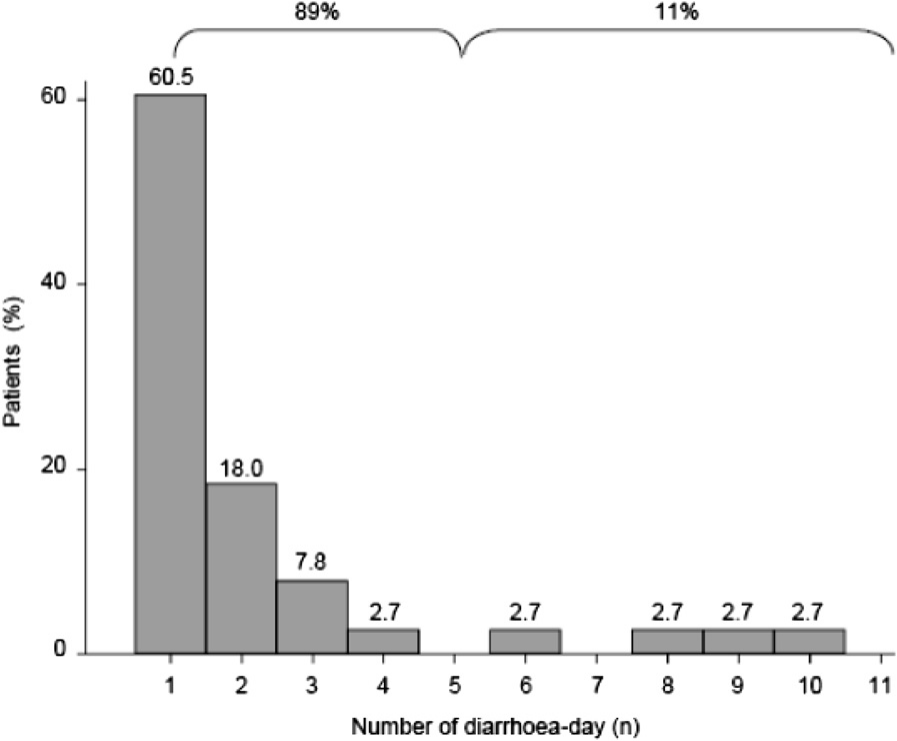
**Number of diarrhoea days in patients with at least 1 day of diarrhoea.** The number of diarrhoea days were measured in patients with at least 1 day of diarrhoea in the first 14 days in the ICU. The first 24 hours in the ICU were not analysed. Diarrhoea was defined as at least three liquid stools per day. The percentage of patients with 1 to 10 diarrhoea days is indicated at the top of bars. The percentage of patients with ≤4 and >4 diarrhoea days was 89 and 11, respectively.

During the 14 first ICU days, at least 1 day of diarrhoea was observed in 14% of patients (Tables 1 and 2). Among the 278 included patients, 42 were sought for a *C. difficile* infection at least once during their ICU stay, but the infection was diagnosed in only two (overall incidence of 0.7%). Out of 1,595 patient-days, 83 were associated with diarrhoea: the diarrhoea incidence rate was 5.2 per 100 patient-days (Table 2). The median day of diarrhoea onset was the sixth day after admission. Table 2 shows the median number of diarrhoea incidents per patient, the median number of liquid stools per diarrhoea, and the median day of diarrhoea onset during the 14 first days in the ICU. Figure 2 reports the number of days with diarrhoea per patient. In 89% of patients, ≤4 diarrhoea days were observed during the ICU stay.

**Table 2:** Epidemiology of diarrhoea during the 14 first days of the ICU stay

**Variable**	**Units**	**Data**
Diarrhoea incidence	Diarrhoea days (per 100 patient–days)	83 (5.2)
Number of patients with diarrhoea	*n* (%)	38 (14)
Number of diarrhoea days per patient	Median (range)	1 (1; 10)
Number of liquid stools per diarrhoea	Median (range)	4 (3; 16)
Days of diarrhoea onset	Median (range)	6 (2; 14)

Diarrhoea was defined as at least three liquid stools per day. Diarrhoea incidence is reported as patient-days by the total of the 1,595 analysed patient-days.

### Diarrhoea risk factors

A multivariate analysis using Generalized Estimated Equations logit regression was performed to determine the diarrhoea risk factors. The presence of EN *per se* had no impact on the risk of diarrhoea (relative risk = 0.87 (0.46 to 1.66)). However, EN, when delivering more than 60% of energy target, increased the risk of diarrhoea by 1.75 (1.02 to 3.01). This effect was not influenced by gender (data not shown). The other factors significantly and independently associated with the risk of diarrhoea were antibiotics and antifungal drugs (Table 3). The highest relative risk was observed with antibiotics. SAPS II, BMI, immunosuppressants, and prokinetics were not associated with the risk of diarrhoea (Table 3).

**Table 3:** Diarrhoea risk factors

	**Exposure**	**Number of diarrhoea days on exposure time**	**Incidence rate**	**Estimated relative risk**^**a**^	
	**(patient-days)**		**(per 100 patient-days)**	**95% CI**	***P *** **value**
Baseline characteristics					
SAPS II >48 vs. ≤48	702	39	5.56	1.48 (0.64; 3.41)	0.356
BMI ≥25 kg/m^2^ vs. <25 kg/m^2^	760	39	5.13	0.77 (0.30; 1.97)	0.595
Risk factor exposure					
EN >60% vs. ≤60% energy target	835	59	7.07	1.75 (1.02; 3.01)	0.042
Antibiotics: yes vs. no	817	73	8.94	3.64 (1.26; 10.51)	0.017
Antifungal drugs: yes vs. no	142	36	25.35	2.79 (1.16; 6.70)	0.022
Immunosuppressants: yes vs. no	69	24	34.78	1.95 (0.57; 6.71)	0.287
Prokinetics: yes vs. no	63	4	6.35	1.44 (0.28; 7.38)	0.659

Diarrhoea was defined as at least three liquid or soft stools per day. The exposure to risk factors was present if the factor was observed during the 48 hours preceding a diarrhoea day. BMI, body mass index; CI, confidence interval; EN, enteral nutrition; SAPS, Simplified Acute Physiology Score. ^a^Relative risk estimated with Generalized Estimated Equations logit regression.

A stratified analysis was conducted to analyse the risk of diarrhoea under exposure to a combination of EN covering more than 60% of the energy target and the administration of antibiotics or antifungal drugs. This analysis showed that antibiotics and antifungal drugs increased the diarrhoea incidence whatever the coverage level of the energy target (Table 4). In patients receiving more than 60% of the energy target by EN in combination with antibiotics or antifungal drugs, the diarrhoea incidence was fivefold increased in comparison with patients receiving more than 60% of the energy target by EN without antibiotics or antifungal drugs.

**Table 4:** Impact of the combination of enteral nutrition with antibiotics or antifungal drugs on diarrhoea incidence

	**Exposure**	**Number of diarrhoea days on exposure time**	**Incidence rate**	**Incidence rate ratio**^**a**^
	**(patient-days)**		**(per 100 patient-days)**	**(95% CI)**
EN ≤60% energy target				
Absence of antibiotics	169	4	7.6	3.2 (1.1; 12.9)
Presence of antibiotics	264	20		
EN >60% energy target				
Absence of antibiotics	295	6	9.8	4.8 (2.1; 13.7)
Presence of antibiotics	540	53		
EN ≤60% energy target				
Absence of antifungal drugs	353	17	22.2	10.6 (4.3; 27.4)
Presence of antifungal drugs	54	12		
EN >60% energy target				
Absence of antifungal drugs	822	30	27.3	5.0 (2.8; 8.7)
Presence of antifungal drugs	88	24		

The exposure to risk factors was present if the factor was observed during the 48 hours preceding a diarrhoea day. Diarrhoea was defined as at least three liquid stools per day. CI, confidence interval; EN, enteral nutrition. ^a^Stratified analysis: incidence rate ratio adjusted with Mantel–Haenszel weights.

## Discussion

This study shows that 14% of patients presented at least 1 day of diarrhoea during the first 2 weeks in the ICU. The diarrhoea risk factors are the use of antibiotics and antifungal drugs. EN is a diarrhoea risk factor only if EN is covering >60% of the energy target. The combination of EN covering >60% of the energy target with antibiotics or antifungal drugs increases diarrhoea incidence, and should be considered the main diarrhoea risk factor. These results clearly suggest that EN, even if covering >60% of the energy target, should not be incriminated as the sole cause of diarrhoea in the ICU.

The prevalence of diarrhoea in ICU patients has been previously reported from 2 and 95% [10]. This variation is explained by different methodologies. In our study, the World Health Organisation definition of diarrhoea was chosen [1]. Because weighing stools in the routine practice of ICU is practically almost impossible, defining diarrhoea as at least three liquid stools per day appears to be the most applicable criteria in daily practice and the best reflection of ICU caregivers’ workload induced by diarrhoea. Our incidence of diarrhoea (14%) is lower than that reported by others [3,22]. In the study by Jack and colleagues, diarrhoea was reported in 78% of a subgroup of patients with continuous enteral feeding [3], whereas patients with parenteral nutrition or oral feeding were not excluded from our study. By including only patients with EN and other specific criteria, the study by Jack and colleagues has selected a subgroup of patients at higher risk of diarrhoea. However, our study certainly underestimated the overall risk of diarrhoea in the ICU, since the occurrence of diarrhoea was limited to the first 14 days in the ICU, and the first 24 hours in the ICU were excluded from the analysis. However, our results are comparable with other studies performed in medical and surgical ICUs, which found a prevalence of diarrhoea of 9% [5,8]. The design of the study by Montejo was close to ours: prospective inclusion of 400 patients during a consecutive period of 1 month [5,8].

Based on older studies [23,24], *C. difficile* infection is always suspected when diarrhoea occurs in the ICU, mainly in patients with antibiotics [25]. In our tertiary ICU, a low incidence of *C. difficile* infection (0.7%) was observed. This is in accordance with studies that found no *C. difficile* infection in cohorts of 39 and 72 ICU patients with diarrhoea, respectively [3,26]. These results indicate that *C. difficile* infection is nowadays a rare cause of diarrhoea in the ICU. However, because of its potentially severe consequences and transmission, *C. difficile* infection has to be sought in case of diarrhoea in ICU patients.

In this study, antifungal or antibiotics drugs were identified as independent risk factors for diarrhoea. However, the analysis of the contribution of the different classes of antibiotics was not possible because the study samples were too small. This limitation has to be addressed in future studies.

EN is a diarrhoea risk factor only when the EN is delivering >60% of the energy target. This clearly suggests that the EN volume, outflow, and the amount of delivered energy may play a role. Early EN constitutes the first choice of nutritional support in the ICU when the gastrointestinal tract is functional [11]. This recommendation is supported by the presumed beneficial effects of EN on the intestinal trophicity and epithelial intestinal barrier, and clinical outcome [27], and by its lower material-related costs in comparison with parenteral nutrition [28]. The role of EN in the onset of diarrhoea has long been suspected [29], but a recent meta-analysis did not suggest an increased diarrhoea risk with EN as compared with parenteral nutrition [30]. Because other factors could increase the diarrhoea risk, diarrhoea onset in ICU patients treated with EN must not be systematically considered a nonfunctionality of the gastrointestinal tract and should not lead to the systematic discontinuation of EN. The reduction or discontinuation of EN would increase the risk for EN of protein-energy deficit [31,32], which is associated with an increased complication rate [15,33]. Recently, a randomised controlled trial indicated that preventing energy deficit with supplemental parenteral nutrition could decrease the rate of infections in ICU patients with EN failure [18], but its impact on the risk of diarrhoea has never been studied. One could hypothesise that by limiting the energy coverage by EN, supplemental parenteral nutrition could decrease the risk of diarrhoea. Nevertheless, in the absence of interventional clinical studies, the management of diarrhoea is still based on nonvalidated protocols [2]. To date, the best management of EN-associated diarrhoea is its prevention, based on the respect of EN initiation and administration rules. If EN is considered the primary cause of diarrhoea, changes in the administration flow rate or replacement of the EN solution can be considered. As only fibre-enriched EN formulas were used in this study, the effect of delivering more than 60% of the energy target with nonfibre-enriched formulas could not be determined.

In our study, patients with diarrhoea were most frequently women and had lower BMI. This would have been due to the fact that females, because of their lower weight, and patients with lower BMI are more frequently covering more than 60% of their energy target. However, BMI and female gender were not found to be confounding factors for diarrhoea risk. As reported by others [3,5,22], patients with diarrhoea had higher disease severity scores at admission and a higher ICU length of stay. However, our study does not allow the conclusion that diarrhoea is associated with worse clinical outcome. Indeed, the putative confounding factors were not studied, since this was beyond the scope of the study. Nevertheless, the higher proportion of patients with bedsores in the diarrhoea subgroup strongly suggests that diarrhoea could have an impact on the risk of complications [34], ICU caregivers’ workload, and costs.

## Conclusion

The incidence of diarrhoea is 14% during the first 2 weeks in a mixed population of patients in a tertiary referral ICU. Delivering more than 60% of the energy target by EN, antibiotics, and antifungal drugs are diarrhoea risk factors. Diarrhoea risk is further increased when EN covering more than 60% of the energy target is combined with antibiotics or antifungal drugs. Studies are needed to better understand diarrhoea physiopathology when EN and antibiotics or antifungal drugs are combined in ICU patients in order to optimise the clinical care and cost management.

## Key messages

• EN is a diarrhoea risk factor only when covering at least 60% of the energy target.

• The combination of antibiotics or antifungal drugs with EN delivering more than 60% of the energy target markedly increased the risk of diarrhoea.

• Of ICU patients, 14% experience at least 1 day of diarrhoea.

• Of diarrhoea episodes, 89% last 4 days or less.

• *C. difficile* infection was an infrequent cause of diarrhoea: incidence of 0.7%.

## Abbreviations

BMI: Body mass index; EN: Enteral nutrition; SAPS: Simplified acute physiology score.

## Competing interests

RT has received consultancy fees from Baxter, B Braun, Nestlé Medical Nutrition, and Nutricia and research grants from Fresenius-Kabi. CP has received research grants and consulting fees from Abbott, Baxter, B Braun, Cosmed, Fresenius Kabi, Nestlé Medical Nutrition, Novartis, Nutricia-Numico, Pfizer, and Solvay. SG, AC, ND and CPH declare that they have no conflicts of interest.

## Authors’ contributions

RT conceived the study, participated in its design, collected, analysed and interpreted the data, and drafted the manuscript. SG participated in the study design, collected, analysed and interpreted the data, and drafted the manuscript. AC analysed and interpreted the data, and drafted the manuscript. ND collected the data. CPH participated in the study design, interpreted the data, and drafted the manuscript. CPH conceived the study, participated in its design, analysed and interpreted the data, drafted the manuscript and obtained the funding. All authors read and approved the final manuscript.
